# A Thermal and Enzymatic Dual‐Stimuli Responsive DNA‐Based Nanomachine for Controlled mRNA Delivery

**DOI:** 10.1002/advs.202204905

**Published:** 2022-12-03

**Authors:** Feng Li, Xiaolei Sun, Jing Yang, Jin Ren, Mengxue Huang, Shengqi Wang, Dayong Yang

**Affiliations:** ^1^ Frontiers Science Center for Synthetic Biology Key Laboratory of Systems Bioengineering (MOE) Institute of Biomolecular and Biomedical Engineering School of Chemical Engineering and Technology Tianjin University Tianjin 300350 P. R. China; ^2^ Beijing Institute of Microbiology and Epidemiology Beijing 100850 P. R. China

**Keywords:** DNA nanotechnology, mRNA delivery, nanomedicine, self‐assembly

## Abstract

The extreme instability of mRNA makes the practical application of mRNA‐based vaccines heavily rely on efficient delivery system and cold chain transportation. Herein, a DNA‐based nanomachine, which achieves programmed capture, long‐term storage without cryopreservation, and efficient delivery of mRNA in cells, is developed. The polythymidine acid (Poly‐T) functionalized poly(*N*‐isopropylacrylamide) (DNA‐PNIPAM) is synthesized and assembled as the central compartment of the nanomachine. The DNA‐PNIPAM nano‐assembly exhibits reversible thermal‐responsive dynamic property: when lower than the low critical solution temperature (LCST, ≈32 °C) of PNIPAM, the DNA‐PNIPAM transforms into extension state to expose the poly‐T, facilitating the hybridization with polyadenylic acid (Poly‐A) tail of mRNA; when higher than LCST, DNA‐PNIPAM re‐assembles and achieves an efficient encapsulation of mRNA. It is remarkable that the DNA‐PNIPAM nano‐assembly realizes long‐term storage of mRNA (≈7 days) at 37 °C. Biodegradable 2‐hydroxypropyltrimethyl ammonium chloride chitosan is assembled on the outside of DNA‐PNIPAM to facilitate the endocytosis of mRNA, RNase‐H mediating mRNA release occurs in cytoplasm, and efficient mRNA translation is achieved. This work provides a new disign principle of nanosystem for mRNA delivery.

## Graphical abstract

A thermal and enzymatic dual‐stimuli responsive DNA‐based nanomachine by using dynamic assembly of polymer framework and hybridization between DNA and mRNA is reported, achieving programmed capture and release, long‐term storage without cryopreservation, and efficient delivery of mRNA in cells. This work provides a new design principle of nanosystem for mRNA delivery.

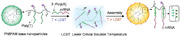

## 1 Introduction

The innovation and application of vaccines opened the door to modern immunology.^[^ ^1^ ^]^ On the other hand, conventional vaccine approaches, such as live attenuated and inactivated pathogens and subunit vaccines, are hindered by the requirement of rapid development and large‐scale deployment for clinical application.^[^ ^2^ ^]^ To overcome these obstacles, nucleic acid vaccines are emerging as promising alternatives to conventional counterparts.^[^ ^3^ ^]^ Especially, the mRNA‐based vaccines distinguish themselves in the vaccine advances by virtue of the superior advantages: mRNA is non‐integrating and can be completely degraded in living cells; thus, avoiding the potential gene integration mutagenesis; mRNA can be translated instantly without requirement for transport across the nuclear membrane which is the way that DNA therapeutically works; what's more, mRNA vaccines have the potential for rapid, inexpensive, and scalable manufacturing via in vitro transcription reactions to cope with the outbreaks of major epidemics such as COVID‐19.^[^ ^4^ ^]^

Nevertheless, mRNA would be quickly degraded by extracellular RNases and can't be internalized efficiently into cells due to its polyanion property.^[^ ^5^ ^]^ To address the transfection obstacles, a range of cationic vectors has been developed for mRNA delivery.^[^ ^6^ ^]^ In recent years, a large body of preclinical data on mRNA vaccines has been accumulated with the development of mRNA delivery technology.^[^ ^7^ ^]^ Regrettably, the inappropriate binding strength between mRNA and cationic vectors often severely affected mRNA cytosolic release and expression efficiency.^[^ ^8^ ^]^ Therefore, the efficient carriers for mRNA delivery should be carefully designed to address the contradiction between binding affinity and cytosolic release of mRNA. However, due to the complex secondary structure of mRNA, designing subtle structures of vectors to achieve controlled assembly and release of mRNA remains a major challenge till now.

The flourishing smart deoxyribonucleic acid (DNA)‐based nanomaterials imply opportunity for the advances in the delivery of mRNA vaccines by virtue of the superiorities of DNA molecules such as unparalleled sequence programmability, precise molecular recognition, and controllability in stimulus responsiveness. Till now, DNA nanomaterials have been widely explored as delivery system of chemotherapeutics and nucleic acid drugs.^[^ ^9^ ^]^ However, due to the complexity–scalability error issues of only DNA‐based materials and complex secondary structure of mRNA, limited DNA nanomaterials have been developed for controlled mRNA delivery.^[^ ^10^ ^]^

**Scheme 1:**
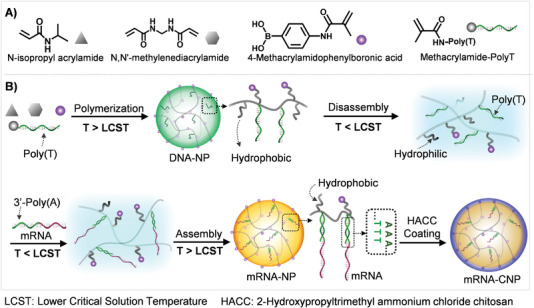
Molecular design and preparation of the nanomachine for mRNA delivery. A). The monomers and their corresponding legends used in the polymerization for the preparation of DNA integrated nanoparticles (DNA‐NPs). B) The synthesis and thermal‐responsive phase transition of PNIPAM‐based nanostructure for efficient capture and encapsulation of mRNA to prepare mRNA loaded nanomachine (mRNA‐CNP).

Herein, we developed a thermal and enzymatic dual‐stimuli responsive DNA‐based nanomachine by integrating DNA into a synthetic covalent polymer system, which enabled spatiotemporally programmed capture and release of mRNA; and thus, achieved efficient mRNA delivery in different cells (**Scheme** **1**). The nanomachine was designed as a hierarchical nanostructure with a poly‐*N*‐isopropylacrylamide (PNIPAM) formed core and a biodegradable 2‐hydroxypropyltrimethyl ammonium chloride chitosan formed shell. PNIPAM was a thermal‐responsive polymer with a lower critical solution temperature (LCST) of ≈32 °C in water. In detail, the PNIPAM was highly hydrophilic below LCST and hydrophobic above LCST. When the temperature was higher than LCST, the PNIPAM was hydrophobic; thus, assembling into nanoparticles.^[^ ^11^ ^]^ A poly‐T DNA sequence was designed as the side chain of PNIPAM for mRNA capture. The thermal‐triggered hydrophilic–hydrophobic transition property of PNIPAM endowed the core structure with reversible assembly and disassembly property. When the temperature was below LCST of PNIPAM, the core was dissociated to facilitate the capture of mRNA through base pairing between the poly‐T and poly‐A tail of mRNA. Subsequently, the system was heated to above LCST, and the PNIPAM backbone underwent phase transition to re‐assemble into nano‐assembly, in which mRNA was efficiently encapsulated. Furthermore, a biodegradable 2‐hydroxypropyltrimethyl ammonium chloride chitosan (HACC) was coated on the surface of mRNA‐encapsulated nanostructure to facilitate the cellular uptake and protect the inside mRNA from RNase attack in the body milieu environment. When reaching cytoplasmic environment, chitosan derivative was degraded and the endogenous RNase H that specifically cleaved RNA strand of an RNA–DNA hybrid mediated the release of mRNA to promote an efficient mRNA translation.

## 2 Results and Discussion

**Figure 1:**
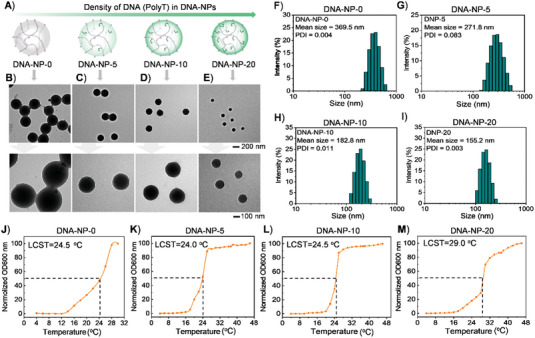
A) Schematic illustration of DNA‐NPs with various densities of poly T side chains, which were denoted as DNA‐NP‐0, DNA‐NP‐5, DNA‐NP‐10, and DNA‐NP‐20, respectively. B–E) The TEM images of DNA‐NP‐0, DNA‐NP‐5, DNA‐NP‐10, and DNA‐NP‐20 with different magnifications. F–I) Hydrodynamic diameter distributions of DNA‐NP‐0, DNA‐NP‐5, DNA‐NP‐10, and DNA‐NP‐20 depending on DLS analysis. J–M) Cyclic turbidity curves of DNA‐NP‐0, DNA‐NP‐5, DNA‐NP‐10, and DNA‐NP‐20.

The DNA/polymer nanoparticle (DNA‐NP) was synthesized via thermal initiating precipitation polymerization of *N*‐isopropylacrylamide (NIPAM), 4‐methacrylamidophenyl‐boronic acid (4‐MAPBA), *N*,*N*′‐Methylenebisacrylamide (Bis), and methacrylamide‐Poly‐T (Acrydite‐Poly‐T) with a similar protocol that we recently reported (Scheme 1A,B).^[^ ^12^ ^]^ In the process, the *N*‐isopropylacrylamide (NIPAM) could be polymerized to form the framework of the nanoparticles, Bis was introduced as covalent cross‐linking, and DNA was tethered as the side chains of the NIPAM polymer chains. The concentrations of NIPAM and 4‐MAPBA in the polymerization reaction were optimized to be 65.5 and 5.6 mm, respectively. The concentration of Bis was vital for the thermal‐responsive assembly of the PNIPAM; when there was no Bis linking in the nanoparticles, the nanoparticles would completely disassemble into free polymers when the temperature was lower than LCST. While, when the temperature was increased to above LCST, no nanoparticles were reformed except for flocculent precipitates. According to our previous report, the concentration of Bis was set as 1.3 mm.^[^ ^12^ ^]^ The number of T was optimized to be 33, and the Tm value between Poly‐T and Poly‐A of mRNA was ≈43 °C, which not only guaranteed the stability of hybrids of Poly‐T and Poly‐A in vivo but also could avoid excessive degradation of the Ploy‐A tail by RNase H which could occur inside cells, in turn resulting in decreased stability of mRNA. The concentrations of Acrydite‐Poly‐T were set as 0 µm, 5 µm, 10 µµ, and 20 µµ, yielding corresponding nanoparticles denoted as DNA‐NP‐0, DNA‐NP‐5, DNA‐NP‐10, and DNA‐NP‐20, respectively (**Figure** **1**A). The morphology and size of the obtained nanoparticles were characterized by transmission electron microscope (TEM) and scanning electron microscope (SEM). TEM images showed homogeneous spherical nanoparticles with diameters of ≈343.2 nm, ≈227.1 nm, ≈197.5 nm, and ≈109.0 nm for DNA‐NP‐0, DNA‐NP‐5, DNA‐NP‐10, and DNA‐NP‐20, respectively (Figure 1B–E). SEM images showed similar diameters of the obtained nanoparticles to those in TEM images (Figure S1, Supporting Information). The hydrodynamic diameters of the nanoparticles were analyzed with dynamic light scattering measurement (DLS). The DLS results showed hydrodynamic diameters of 369.5, 271.8, 182.8, and 155.2 nm for DNA‐NP‐0, DNA‐NP‐5, DNA‐NP‐10, and DNA‐NP‐20, respectively. These analysis results demonstrated that the introduction of DNA could decrease the diameters of the hybridized nanoparticles (Figure 1F–I). It was speculated that the addition of hydrophilic DNA had important influence on the hydrophilic–hydrophobic property of the polymerization reaction system, nucleation process, and crosslinking process; thus, leading to different particle diameters of DNA‐NPs.

As aforementioned, the PNIPAM polymer backbone of DNA‐NPs had a LCST in aqueous solution. When the temperature was lower than the LCST of PNIPAM, PNIPAM polymer became hydrophilic, which could cause disassembly of the nanoparticles (Scheme 1B). To explore the potential of DNA‐NPs and mRNA protection, the LCST of the DNA‐NPs was measured. To test the LCST of the DNA‐NPs, turbidity analysis was performed on the DNA‐NPs with UV–vis absorption spectrum at varied temperatures. The absorption of DNA‐NPs at 600 nm was collected. For each sample, the absorption value was normalized and plotted (Figure 1J–M). As reported, the temperature corresponding to half of the maximum absorbance value was defined as LCST. The results showed that DNA‐NP‐0, DNA‐NP‐5, and DNA‐NP‐10 have a similar LCST of ≈24.0–24.5 °C; while, the LCST of DNA‐NP‐20 was ≈29.0 °C. The LCST was largely dependent on the hydrogen‐bonding capabilities of the constituent monomer units in the polymer. The hydrogen bonding of the basic sites on the polymer with water favors dissolution. Consequently, increasing DNA side chains on the PNIPAM polymer resulted in an elevated phase transition temperature. Therefore, DNA‐NP‐20 exhibited a higher LCST than the other three nanoparticles. The absorbances of DNA‐NP‐5, DNA‐NP‐10, and DNA‐NP‐20 were greatly increased and reached a high steady value when the temperature was higher than 16 °C. Take the DNA‐NP‐20 as an example, the hydrazination size was continuously reduced when the temperature of the solution was increased from 4 °C to 45 °C, indicating that the polymer nanoparticles were in the deswelling state at body temperature, which facilitated protection of the packaged mRNA against enzymes attack (Figure S2A, Supporting Information). Moreover, TEM images of DNA‐NP‐20 showed that reassembled nanoparticles had similar size and morphology to the initial ones (Figure S2B, Supporting Information).

**Figure 2:**
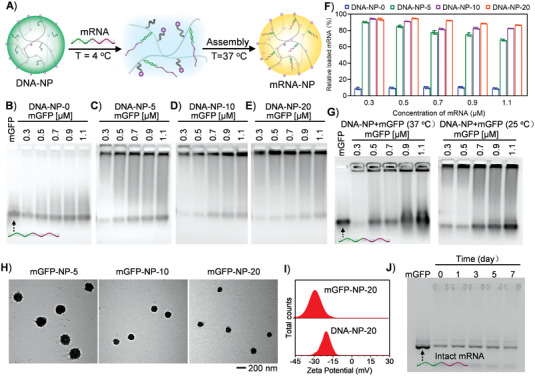
Characterization of mGFP assembly in DNA‐NPs. A) Schematic illustration of the temperature meditating disassembly and assembly of DNA‐NP for mGFP capture and encapsulation. B–E) Gel electrophoresis analysis of mGFP capture capability for DNA‐NP‐0, DNA‐NP‐5, DNA‐NP‐10, and DNA‐NP‐20 versus different mGFP concentrations according to the strategy represented in (A). F) Quantitative analysis of the loaded mGFP versus corresponding total mGFP concentrations in (B,C,D,E) using image J software. Data represented means ± SD, *n* = 3. G) Gel electrophoresis analysis of mGFP capture capability for DNA‐NP‐20 at constant temperatures of 25 °C and 37 °C. H) The TEM images of mGFP‐NP‐5 (mGFP loaded DNA‐NP‐5), mGFP‐NP‐10 (mGFP loaded DNA‐NP‐10), and mGFP‐NP‐20 (mGFP loaded DNA‐NP‐20). I) The zeta potential of DNA‐NP‐20 and mGFP‐NP‐20. J) The long‐term storage stability of mRNA in mGFP‐NP‐20 without cryopreservation.

mRNA has a complex secondary structure; and therefore, controllable capture of mRNA remains challenging. Till now, mRNA encapsulation in nanocarriers was mainly dependent on electrostatic interaction, which was in poor controllability. For effective capture and encapsulation of mRNA in the nanoparticles, the nanoparticles were first cooled to 4 °C (low temperature commonly used for biological samples, which was below the LCST of DNA‐NPs) to facilitate the disassembly of DNA‐NPs; thus, exposing the DNA poly‐T in the solution for effective hybridization with the poly‐A tail of mRNA. Afterward, the temperature was increased to 37 °C; and thus, the PNIPAM polymer backbone increased its hydrophobicity and re‐assembled into nanoparticles to achieve effective encapsulation of already captured mRNA. In our work, mRNA that could express enhanced green fluorescent protein (EGFP) was adopted as the research model. According to the procedure detailed in **Figure** **2**A, the mRNA capture capability of series of DNA‐NPs was tested. DNA‐NP‐0, DNA‐NP‐5, DNA‐NP‐10, and DNA‐NP‐20 were cooled to 4 °C and incubated with different concentrations of EGFP mRNA (mGFP) for 30 min. The capture efficiency was characterized with agarose gel electrophoresis (Figure 2B–E). In the electrophoresis gels, the mRNA captured by DNA‐NPs was trapped in the wells and was quantitatively analyzed (Figure 2F). The results showed that after incubation with DNA‐NP‐0, almost all mGFP remained free in the samples and less than 10% of mGFP was trapped in the wells with mGFP concentrations ranging from 0.3 to 1.1 µm, demonstrating little mGFP was captured by DNA‐NP‐0. In contrast, for DNA‐NP‐5, DNA‐NP‐10, and DNA‐NP‐20, most mGFP molecules were trapped in the wells of gels after co‐incubation, showing the efficient capture of mGFP in these nanoparticles. The difference indicated that the binding between mRNA and DNA‐NP was realized by base pairing between the capture chain Poly‐T and 3’Poly‐A tail of mRNA, rather than physical entanglement. With the increasing concentration of mGFP, the percentage of mRNA trapped in the wells decreased for DNA‐NP‐5, DNA‐NP‐10, and DNA‐NP‐20. However, when the concentration of mGFP was set as high as 1.1 µm, the percentage of mGFP trapped in wells of gel was still more than 86.6%, confirming the high mGFP capture capability for DNA‐NP‐20. The gel electrophoresis results demonstrated that the DNA‐NPs with higher Poly‐T density had better performance in mRNA loading, especially in the case of high concentration of mRNA. Therefore, DNA‐NP‐20 was selected for subsequent investigations, considering its proper LCST and good mRNA capture capability.

To confirm that the phase transformation of DNA‐NPs could facilitate the mRNA capture, DNA‐NP‐20 was incubated with mRNA at room temperature (25 °C) and physiological temperature (37 °C), respectively, which was followed by gel electrophoresis analysis (Figure 2G). The results showed that, when mGFP concentrations were set as 0.7, 0.9, and 1.1 µm, respectively, the capture efficiencies were decreased to 62.1%, 55.9%, and 49.1% at 25 °C, respectively, and were further decreased to 65.3%, 34.6%, and 31.8% at 37 °C, respectively (Figure S3, Supporting Information). It was proposed that, at 25 °C, a fraction of *N*‐isopropyl groups on PNIPAM backbone were transformed from hydrophobicity to hydrophilicity; and thus, DNA‐NP‐20 maintained an intermediate state between disassembly and assembly. At 37 °C, the PNIPAM backbone in DNA‐NP‐20 was completely separated from the aqueous phase. Consequently, capture strands (Poly‐T) were enclosed in DNA‐NP‐20 at both 25 °C and 37 °C, leading to an insufficient interaction with poly‐A tail of mGFP; and thus, a poor mGFP capture capability due to steric‐hindrance effect.

The effect of incubation time on the capture efficiency of mGFP was tested. DNA‐NP‐20 was incubated with mGFP (0.5 µm) for different time periods (0, 5, 10, 20, and 30 min) at 4 °C and then heated to 37 °C followed by analysis with 1% agarose gel electrophoresis. Incubation for 0 min meant that DNA‐NP‐20 was well mixed with mGFP and then heated immediately We found that the 0‐min sample showed similar quantity of trapped mGFP in the wells to the other four samples, demonstrating that the hybridization between the capture chain Poly‐T of DNA‐NP‐20 and Poly‐A of mGFP could hybridize quickly (Figure S4, Supporting Information). To demonstrate the universal applicability, we explored the capture capacity of DNA‐NP‐20 with FLuc mRNA that contained 1929 nucleotides. The results showed a rapid capture capability of DNA‐NP‐20 for FLuc mRNA. The quantitative analysis showed that the capture ratio was ≈60%, which was lower than that for GFP mRNA (Figure S5, Supporting Information). It was proposed that the longer the mRNA, it could encounter more steric hindrance in the assembly process, which could weaken the capture assembly efficiency. Next, the morphology and size of mGFP loading DNA‐NP (mGFP‐NP) were characterized with TEM. The TEM images showed that mGFP‐NP‐5, mGFP‐NP‐10, and mGFP‐NP‐20 were monodispersed with diameters of ≈224, ≈136, and ≈118 nm, respectively (Figure 2H). Considering the high loading efficiency and favorite diameter, mGFP‐NP‐20 was chosen for the following investigations. To further confirm the mGFP loading in the DNA‐NP‐20, zeta potential analysis was performed to explore the potential change of the nanoparticle surface, and the results showed zeta potentials changed from ≈ −20 mV to ≈ −28 mV for DNA‐NP‐20 and mGFP‐NP‐20, respectively (Figure 2I). The significant change in zeta potential demonstrated that the mGFP was successfully assembled in DNA‐NP‐20.

The high susceptibility of mRNA to nuclease‐mediated degradation in physiological environments was the major obstacle to the widespread in vivo applications of mRNA therapeutics. Protecting mRNA from degradation in plasma was vital for practical application of mRNA therapeutics. To test whether the stability of mRNA was improved in the DNA‐NPs, the mGFP‐NPs (final mRNA concentration of 0.42 µm) were incubated in 10% FBS for different time points and then analyzed with agarose gel electrophoresis (Figure S5, Supporting Information). Naked mGFPs incubated in serum were used as controls. The gray values of mRNA trapped in the loading wells were almost unchanged after 4 h incubation, suggesting the DNA‐NPs successfully protected the mRNAs from RNase attack in the serum. In contrast, naked mRNA in the mGFP‐NP samples and control samples underwent fast degradation within 15 min incubation in serum, which showed smeared bands with higher mobility than the intact mRNAs in the gels. The stability of mRNAs enveloped in DNA‐NPs was easily explained by the shielding effect of PNIPAM network. Moreover, as previous literature reported,^[^ ^13^ ^]^ we also tested the thermal stability of mRNA‐NPs under storage without cryopreservation, considering the concern of ultra‐cold storage need of mRNA molecules, which may severely limit the global distribution of mRNA vaccines. The agarose gel electrophoresis image showed that at even a harsh temperature (37 °C), the bands of intact mRNA after 1‐day, 3‐day, 5‐day, and 7‐day storage remained almost identical compared with that of intact mRNA free of 37 °C storage (Figure 2J). The result indicated that the fabricated nanomachine could provide more possible mRNA delivery approaches with more permissive storage conditions before administration, which allowed the packaged mRNA molecules to be intact as long as 7 days under harsh temperature condition. However, when the time was prolonged to more than 12 days, more mRNA was degraded (Figure S6, Supporting Information).

**Figure 3:**
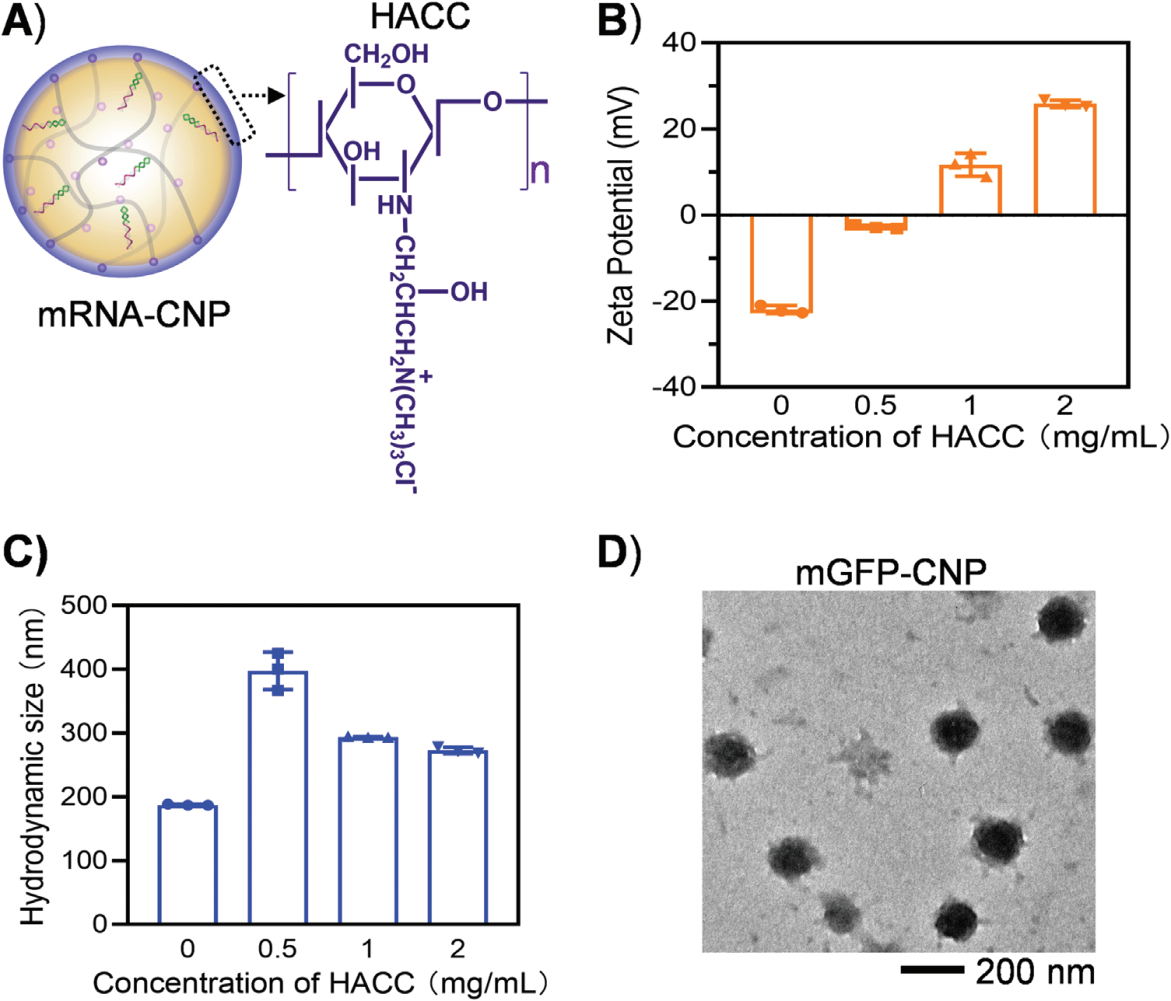
Coating of quaternary chitosan (HACC) on the mRNA‐NP‐20. A) Scheme illustration of HACC coated mRNA‐NP‐20 (mRNA‐CNP) and the molecular formula of HACC. B,C) The changes of zeta potential and hydrodynamic diameters of mGFP‐NP‐20 after coating with HACC. Data represented means ± SD, *n* = 3. D) The TEM image of mRNA‐CNP.

Although the acceptable mRNA loading capability and thermostability have been achieved, the interactions between the nanomachine and cells needed to be considered in order to effectively action in the biological environment. The zeta potential of mGFP‐NP‐20 was close to −30 mV, which could not facilitate the interaction with negatively charged cell membrane; thus, possibly leading to attenuated transfection of mRNA. Therefore, a biofunctionalized shell was introduced in our nanomachine. Chitosan was a natural cationic polysaccharide obtained by deacetylation of chitin.^[^ ^14^ ^]^ Owing to good biodegradability and biocompatibility, chitosan has been widely applied in biomaterials.^[^ ^15^ ^]^ Moreover, chitosan could provide immune adjuvant activity, which was beneficial to the improvement of vaccine efficacy. Considering that the poor solubility of chitosan at physiological pH largely hindered its application in biomedicine, 2‐Hydroxypropyltrimethyl ammonium chloride chitosan (HACC) with improved hydrophilicity was adopted for coating of mGFP‐NP‐20 to obtain mGFP‐CNP to protect the mRNA inside from nuclease attack and to enhance cellular uptake (**Figure** **3**A). HACC was assembled on the mGFP‐NP via electrostatic interaction. When the concentrations of HACC were set as 0.5, 1, and 2 mg mL^−1^, the zeta potentials of the mGFP‐CNPs were −2.75, 11.72, and 25.83 mV, respectively; the average hydrodynamic diameters of mGFP‐CNPs were 397.7, 293.9, and 272.9 nm, respectively (Figure 3B,C; Figure S7, Supporting Information). It was noteworthy that when the concentration of HACC was set as 0.5 mg mL^−1^, the average hydrodynamic diameter was nearly 400 nm presumably due to the aggregation effect of nanoparticles with neutral potential. According to the results of zeta potentials and hydrodynamic diameters, the coating concentration of HACC was set as 1 mg mL^−1^ for the preparation of mGFP‐CNPs in the following studies. The corresponding TEM image of the optimized mGFP‐CNPs showed monodisperse and spherical nanoparticles with diameter of ≈154 nm, which was favorable for the cellular uptake (Figure 3D).

To evaluate the biocompatibility of nanocarrier CNPs, standard MTT (3‐(4,5‐dimethylthiazol‐2‐yl)‐2,5‐diphenyltetrazolium bromide) assay on different cell lines was performed. DC2.4 cells, RAW264.7 cells, Hela cells, and HEK‐293T cells were selected to be incubated with CNPs in varied concentrations for 24 h, respectively. The results showed that in the tested concentrations of CNPs, all the CNPs treated groups showed high viabilities that were comparable to the control groups for the four cell lines, demonstrating the high biocompatibility of CNPs (Figure S8, Supporting Information).

Dendritic cells (DCs) were adopted as a representative cell line to evaluate the cellular uptake of mGFP‐CNPs. To facilitate detection of particle uptake, the Poly‐T in mGFP‐CNPs was labeled with TAMRA (denoted as mGFP‐TAMRA‐CNPs). The CLSM images of the DCs showed increasing fluorescence signals along with the prolonged incubation time with mGFP‐TAMRA‐CNPs (Figure S9, Supporting Information). To quantify the mRNA uptake efficiency, the Cy5 labeled mRNA molecules were packaged in the CNPs to prepare Cy5‐mGFP‐CNPs for flow cytometry (FCM) analysis. The FCM analysis results also showed increasing fluorescence intensity of DCs with prolonging incubation which was consistent with the CLSM images, and 87.86% cells were successfully transfected with Cy5‐mGFP‐CNPs after 4 h incubation (Figures S10 and S11, Supporting Information).

To evaluate the lysosomal escape capability of mGFP‐CNPs, CLSM analysis was performed on Cy5‐mGFP‐CNPs treated DC2.4 cells. DC2.4 cells were incubated with Cy5‐mGFP‐CNPs for 1 h, followed by the further culture of 2, 4, and 6 h in fresh medium, respectively. For CLSM imaging, the lysosomal compartments and nucleus were stained with LysoTracker (green) and DAPI (blue), respectively. The co‐localization of red signals and green signals was observed within 2 h, indicating a lysosomal endocytosis pathway of mGFP‐CNPs. After culture of 4 and 6 h, separated red fluorescence and green fluorescence were observed in the cells as indicated by the white arrows, demonstrating the sustained escape of Cy5‐mGFP‐CNPs from lysosomes into cytoplasm (Figure S12, Supporting Information). Then, to observe the release behavior of mGFP from CNPs, the DC2.4 cells were incubated with Cy5‐mGFP loaded FAM‐CNPs at 37 °C for 1 h, and then, the medium was replaced with fresh medium following by a 6 h incubation for CLSM imaging (Figure S13, Supporting Information). As indicated by the red arrows, there was some obvious separation between the green fluorescence (FAM‐Poly‐T) and the red fluorescence (Cy5‐mRNA), demonstrating that the mGFP was released from the nanopartciles.

**Figure 4:**
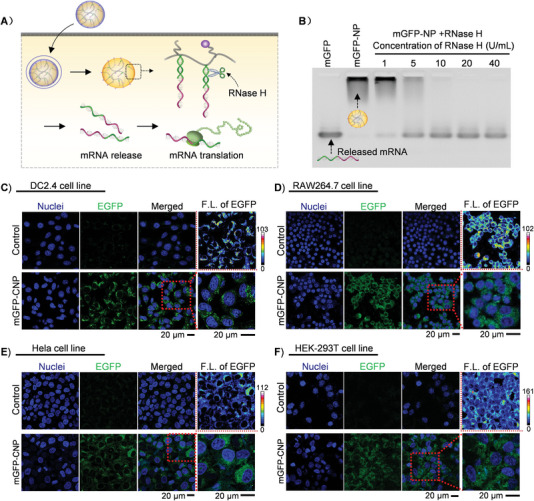
A) The scheme of cellular uptake, mRNA release, and translation of mGFP‐CNP. B) The RNase H mediated mGFP release. C–F) Transfection of mGFP‐CNP in different cell lines including DC2.4 cell line, RAW264.7 cell line, Hela cell line, and HEK‐293T cell line.

The rapid release of mRNA in cytoplasm was vital for efficient transfection. For electrostatically complexed mRNAs, controlled release of mRNA was difficult to achieve in cytoplasm with unclear mechanism. After cellular uptake, the HACC was proposed to be degraded by breaking glycosidic bonds in acidic lysosome; in addition, a variety of enzymes such as protease and glycosidase in lysosome could rapidly degrade chitosan into oligomers with low molecular weight, which was beneficial to the release of the mRNA‐NPs into the cytoplasm.^[^ ^16^ ^]^ Further, the Endogenous ribonuclease H (RNase H) that specifically cleaves RNA in DNA‐RNA hybridization has become a simulating element of interest in intracellular RNA delivery. In our system, mRNA hybridized with Poly‐T DNA. It was prospected that the mRNA could be released from the DNA‐NPs in the presence of RNase H (**Figure** **4**A). To examine the RNase H‐responsive release of mRNA, the mGFP‐NPs were incubated with various concentrations of RNase H (1, 5, 10, 20, and 40 U mL^−1^), followed by gel electrophoresis analysis (Figure 4B). The results showed that when the enzyme activity of RNase H was 1 U mL^−1^, a small amount of mGFP was released; when the enzyme activity went up to 5 U mL^−1^, considerable amounts of mGFP were released; when RNase H reached 20 U mL^−1^, mGFP was completely released from DNA‐NPs within 30 min at 37 °C. After confirming the release of mRNA in the presence of RNase H, to test whether the change of polyA tail affected the translation performance of mGFP, mGFP‐NPs were incubated with RNase H (20 U mL^−1^, the aforementioned concentration enabling the full release of mRNA) to obtain the polyA partially degraded EGFP mRNA, and then, we used these mRNAs to transfect into DC2.4 cells with Lipofectamine 3000. Fluorescence microscope imaging analysis showed significant green fluorescence in DC2.4 cells, demonstrating the successful translation of mGFP (Figure S14, Supporting Information). The result demonstrated that the RNase H‐induced change of poly‐A tail did not show significant adverse effects on the transfection of mGFP.

Furthermore, the applicability of mGFP‐CNPs for in vitro mRNA delivery was tested by incubating mGFP‐CNPs with immune cell line DC2.4 cells. CLSM imaging was carried out to evaluate the mGFP translation effect. The CLSM results showed that after incubation with mGFP‐CNPs for 24 h, significant green fluorescence was observed in DC2.4 cells, confirming the successful mGFP translation. What's more, the fluorescence intensity of mGFP‐CNPs treated DC2.4 cells showed to be ≈4.4 times and 1.7 times stronger than that of naked mRNA treated cells and mGFP‐NPs treated cells, respectively, confirming the HACC‐coating‐mediated enhancement of mGFP transfection (Figure S15, Supporting Information; Figure 4C). Moreover, the transfection capability of mGFP‐CNPs in other three cell lines, including RAW264.7 cell line (another immune cell line), HeLa cell line (cervical cancer cell line), and HEK‐293T cell line (embryonic kidney cell line), were also observed and imaged by CLSM (Figure 4D–F). The CLSM results showed significant green fluorescence signal in all the three cell lines, demonstrating successful transfection of mGFP. The transfection of mGFP‐CNPs could effectively be achieved in multiple cell lines, providing experimental basis for more comprehensive application in vivo of mRNA nanocomplex in the treatment of diseases. It is noteworthy that both DC2.4 cells and RAW264.7 cells were hard‐to‐transfect cell types; thus, the successful transfection of mGFP‐CNPs firmly revealed the great potential in the biomedical applications that were related to immunotherapy.

The biodistribution of the Cy5 labeled mRNA‐CNP (Cy5‐mRNA‐CNP) in vivo was explored in BABL/C mice. The intramuscular injection, which was widely applied in vaccination, was employed in our work because of the appealing characteristics of rapid and full absorption and ideal specific immune responses. After 24 h intramuscular administration of Cy5‐mRNA‐CNP, the biodistribution of the Cy5‐mRNA‐CNP was detected with an in vivo imaging system (Figure S16, Supporting Information). Compared with control group, significant signal fluorescence signals were detected at inguinal lymph nodes adjacent to the injection site for Cy5‐mRNA‐CNP, indicating that the Cy5‐mRNA‐CNP could migrate to accumulate as the lymph nodes.

## 3 Conclusion

In summary, we developed a DNA‐hybrid nanomachine for effective mRNA delivery via clever integration of biopolymers (DNA and chitosan derivative) and synthetic polymer (PNIPAM), in which DNA functionalized PNIPAM framework was coated with chitosan derivative. The nanomachine was well designed with thermal and enzymatic dual‐stimuli responsiveness for controllable mRNA delivery. PolyT DNA strands were covalently installed in the temperature‐sensitive PNIPAM framework, acting as capturers to recognize and bind with mRNA molecules. Taking advantage of the dynamic and reversible thermal‐responsiveness of PNIPAM framework, temperature was controlled to help achieve spatiotemporal assembly of the nanomachine. The mRNA with Poly‐A tail could be effectively hybridized with Poly‐T anchored on the nanomachine in a time‐efficient manner when the temperature was below the LCST; at temperature higher than LCST, the PNIPAM framework was reassembled and mRNA were well embedded inside. Importantly, the nanomachine could provide a favorable environment for mRNA storage without strict requirement. The captured mRNA could be released through the specific cleavage of the RNA–DNA hybrids. Moreover, the coating of chitosan derivative further improved the stability and transfection efficiency of the mRNA delivery system. Our work provided a unique avenue for developing delivery vehicle of mRNA, and achieved impressive mRNA loading and protection, controllable mRNA release, and effective transfection inside different cells. We envision that the DNA‐based nanomachine would promote the development of mRNA‐based therapeutic strategy.

## Conflict of Interest

The authors declare no conflict of interest.

## Supporting information

Supporting Information

## Data Availability

Research data are not shared.
