# Supplementary material for: A Thermal and Enzymatic Dual‐Stimuli Responsive DNA‐Based Nanomachine for Controlled mRNA Delivery
Source: Adv Sci (Weinh). 2022 Dec 3;10(4):2204905. doi: 10.1002/advs.202204905 (PMC9896069; doi:10.1002/advs.202204905)
Supplement: Supplementary file 1 — Supporting Information [file ADVS-10-2204905-s001.pdf]

ADVANCED SCIENCE
Open Access

Supporting Information

for *Adv. Sci.*, DOI 10.1002/advs.202204905

A Thermal and Enzymatic Dual-Stimuli Responsive DNA-Based Nanomachine for Controlled mRNA Delivery

Feng Li, Xiaolei Sun, Jing Yang, Jin Ren, Mengxue Huang, Shengqi Wang and Dayong Yang**

Supporting Information

A thermal and enzymatic dual-stimuli responsive DNA-based nanomachine for controlled mRNA delivery

Feng Li, Xiaolei Sun, Jing Yang, Jin Ren, Mengxue Huang, Shengqi Wang, and Dayong Yang**

Experimental Procedures

Chemicals and Materials

N-isopropylacrylamide (NIPAM) was purchased from Tianjin HEOWNS Biochem Technologies LLC. (Tianjin, China). N,N'-methylenediacrylamide (Bis), 4-Methacrylamidophenyl boronic acid (4-MAPBA) were purchased from Energy Chemical Co. Ltd. (Shanghai, China). Ammonium persulfate (APS) was purchased from Solarbio Science & Technology Co., Ltd. (Beijing, China). RPMI 1640 medium, Dulbecco's modified Eagle's medium (DMEM) were purchased from Gibco, Life Technologies. Fetal bovine serum (FBS) was purchased from Excell Biological Technology Co., Ltd. (Shanghai, China). Opti-MEM Reduced Serum Medium and Lipofectamine 3000 Transfection Reagent were purchased from Thermo Fisher Scientific Inc.. Chitosan quaternary ammonium salt (hydroxypropyl trimethyl ammonium chloride chitosan, HACC) was purchased from Shanghai yuanye Bio-Technology Co., Ltd. (Shanghai, China). 3-(4,5-dimethylthiazol-2-yl)-2,5-diphenyltetrazolium bromide (MTT), 4% formaldehyde, 4',6-diamidino-2-phenylindole (DAPI), DNase/RNase-Free Water, polyvinylidene fluoride (PVDF) membrane (pore size: 0.22 μm) were purchased from Solarbio Science & Technology Co., Ltd. (Beijing, China). CleanCap EGFP mRNA and CleanCap FLuc mRNA were purchased from TriLink Bio Technologies (San Diego, U.S.A.). LysoTracker Green DND-26 was purchased from Yeasen Biotechnology Co., Ltd. (Shanghai, China). RNase H was purchased from New England Biolabs, Inc. (Beijing, China). Label it Nucleic Acid Labeling Kit, Cy5 was purchased from Mirus Bio LLC (Madison, U.S.A.). EasyPure RNA Purification Kit was purchased from TransGen Biotech Co., Ltd. (Beijing, China). All the DNA oligonucleotides of Acrydite-PolyT, TAMRA-Acrydite-PolyT, FAM-Acrydite-PolyT were synthesized by Sangon Biotech Co., Ltd. (Shanghai, China) and all were purified by HPLC. The customized sequences of oligonucleotides are listed in **Table S1**.

Methods

Synthesis of DNA-PNIPAM nanoparticles (DNA-NPs).

The synthetic method of DNA-PNIPAM nanoparticles was similar to the protocol we previously reported.[1] Briefly, 65.5 mM NIPAM (N-isopropylacrylamide), 5.6 mM 4-MAPBA (4-methacrylamidophenyl boronic acid), 1.3 mM Bis (N,N'-Methylenebisacrylamide), 0.1 wt% APS (Ammonium persulphate), Acrydite-PolyT and filtered water were added to make up a 1 mL reaction system with continuing N_2 bubbling. The final Acrydite-PolyT concentrations in reaction system were set as 0 μM, 5 μM, 10 μM, 20 μM for preparation of DNA-NP-0, DNA-NP-5, DNA-NP-10, DNA-NP-20, respectively. The reaction system was under thermal initiating precipitation polymerization at 70 °C with gentle magnetic stirring for 30 min. At the end of the polymerization reaction, the appearance of reaction system turned into milk-white turbid emulsion. The synthesized DNA-NPs were collected by centrifugation (8000 rpm) and redispersed in RNase-free water for further use.

Dynamic light scattering measurement.

Samples containing 200 μg/mL DNA-NPs and 4 ng/μL mRNA were used for DLS measurements with Zetasizer Nano ZS90 (Malvern, U.K.). The DLS measurements were conducted at the temperature of 37 ^{o}C with 120 seconds equilibrium time. The temperature-sensitive hydrodynamic size variations of DNA-NP-20 were tested with the temperature ranging from 4 ^{o}C-45 ^{o}C. Each measurement was performed three times in parallel after the equilibrium time at the set temperature.

Transmission electron microscopy (TEM) observation.

The synthesized bulk solution was centrifugated at 8000 rpm for 10 min and diluted 20 times with filtered water at a final concentration of 400 μg/mL. To verify the morphology recoverability of DNA-NPs, the DNA-NP-20 nanoparticles were resuspended in 1×TAE·Mg^{2+} buffer and placed at 4 ^{o}C for 30 min according to the mRNA loading condition, then the sample was heated to 37 ^{o}C for phase transition, the DNA-NP-20 solution before and after 4 ^{o}C treatment were respectively diluted to 400 μg/mL for TEM observation. Transmission electron microscopy (TEM) observation was performed at an acceleration voltage of 120 kV with a JEM-1400 Flash transmission electron microscope (JEOL, Japan).

Scanning electron microscopy (SEM) observation.

The samples were dropped on the silicon wafers fixed on the SEM sample stage and dried in a vacuum oven at 37 °C overnight. Prior to SEM observation, the samples were sprayed with Au coating and then observed using scanning electron microscope (Hitachi-S4800 FESEM, Japan).

LCST measurement of DNA-NPs using turbidity method.

DNA-NP-0, DNA-NP-5, DNA-NP-10, DNA-NP-20 were redispersed in 500 µL filtered water (2 mg/mL). The absorbance of the solutions was measured at 600 nm at increasing temperature points from 4 ^{o}C to 46 ^{o}C using an UV spectrophotometer (Evolution 201 UV-Visible Spectrophotometers, Thermal Fisher Scientific). The heating rate and heating step of heating block were set as 0.5 ^{o}C/min, 2 ^{o}C, respectively. The equilibrium time was set as 5 min. The LCST was defined as the temperature exhibiting 50% of the maximum optical absorbance of the sample solution.

Preparation of mGFP-NP nanocomplexes.

The preparation of mGFP-NP nanocomplexes consisted of 3 consecutive steps: phase transition under LCST (4 ^{o}C) for mRNA capturing, mRNA loading, and phase transition above LCST (37 ^{o}C) for mRNA embedding. The solution of synthesized DNA-NPs was centrifuged at 8000 rpm for 15 min to collect the DNA-NPs deposition, and then the DNA-NPs deposition was redispersed and concentrated in RNase-free 1×TAE·Mg^{2+} buffer by a factor of 3. The concentrated DNA-NPs solution was placed at 4 ^{o}C to ensure sufficient phase transition and swelling. When the appearance of DNA-NP solution became transparent, EGFP mRNA was added to 24 µg/µL DNA-NPs (DNA-NP-0, DNA-NP-5, DNA-NP-10, DNA-NP-20) at the final hybridization concentration gradient of 0.3 µM, 0.5 µM, 0.7 µM, 0.9 µM, 1.1 µM and fully mixed. Then, all samples were placed at 4 ^{o}C for 30-min hybridization. Next, all samples loaded with mRNA were briefly heated to 37 ^{o}C (>LCST) to undergo a phase transition and a morphology recovery.

Serum stability assay.

The nuclease resistance of mGFP-NP was tested by incubating with 10% fetal bovine serum (FBS, ExCell Bio, Shanghai) at 37 ^{o}C over different time length: 15 min, 30 min, 1 h, 2 h, 4 h. The naked mRNA groups were under the same FBS treatment as the mGFP-NP groups.

After incubation with FBS, the samples were analyzed with 1% agarose gel electrophoresis in ice bath. The final mRNA concentration was set as 0.42 μM, and the final DNA-NP-20 concentration was set as 16.8 μg/μL.

RNase H-responsive release of mRNA from DNA-NPs.

50.4 μL mGFP-NP nanocomplex system was prepared with 0.5 μM EGFP mRNA and 20 μg/μL DNA-NP-20. 42 μL was added into a RNase-free eppendorf tube for further experiment, the remaining 8.4 μL was used as non-RNase H treatment control. 6.25 μL of 10× RNase H reaction buffer was added to the mGFP-NP solution, and then RNase-free water was mixed to make up the volume to 62.5 μL. The 62.5 μL mGFP-NP solution was divided equally into 5 groups with a volume of 12.5 μL. Prior to addition, RNase H (New England Biolabs, Beijing) was thawed on ice and diluted with RNase-free water to five concentrations: 50 U/mL, 250 U/mL, 500 U/mL, 1000 U/mL, 2000 U/mL. Afterward, the corresponding diluted RNase H solution was added to the 12.5 μL preformed reaction system and mixed well to obtain the five RNase H-cleavage reaction systems possessing five different enzymatic activities: 1 U/mL, 5 U/mL, 10 U/mL, 20 U/mL, 40 U/mL. Then, the five reaction systems were placed at 37 ^{o}C for 30 min incubation, which were followed by analysis with 1% agarose gel electrophoresis in ice bath.

Evaluation of mRNA loading speed and loading temperature.

To study the mRNA loading speed, EGFP mRNA molecules at a final concentration of 0.5 μM were loaded in DNA-NP-20 at a final concentration of 21.1 μg/μL and Fluc mRNA molecules at a final concentration of 0.11 μg/μL were loaded in DNA-NP-20 at a final concentration of 22.2 μg/μL, the loading time periods were set as 1 min, 5 min, 10 min, 20 min, 30 min. All groups were placed at 4 ^{o}C for different time length. The 0-min group was to mix well the mRNA and the swelling DNA-NP-20, and then immediately to perform the agarose gel electrophoresis. After undergoing different time length of mRNA loading, all groups were mixed with loading buffer for subsequent agarose gel electrophoresis. To assess the impact of temperature on mRNA loading efficiency, two typical temperature values were tested: 25 ^{o}C (Room temperature), 37 ^{o}C (Physiological temperature). The EGFP mRNA loading concentration gradient was set as 0.3 μM, 0.5 μM, 0.7 μM, 0.9 μM, 1.1 μM, respectively. Samples mixed with various concentrations of EGFP mRNA were placed either at 25 ^{o}C or 37 ^{o}C for the entire loading process for 30 min. After mRNA capturing process,

the samples of two groups were used to perform the subsequent 1% agarose gel electrophoresis.

Thermalstability of mGFP-NP nanocomplex at harsh temperature.

The mGFP-NP nanocomplex system was prepared with 0.35 μM EGFP mRNA and 20 μg/μL DNA-NP-20 under RNase-free environment. After a 30-min hybridization process at 4 ^{o}C, five mGFP-NP samples were, respectively, stored for 7, 5, 3, 1, 0 days at 37 ^{o}C before RNase H treatment, which was followed by analysis with 1% agarose gel electrophoresis for evaluation of intact mRNA after storage at harsh temperature.

Agarose gel electrophoresis.

All samples were prepared with addition of loading buffer prior to gel electrophoresis. The gel electrophoresis was performed using 1% (w/w) agarose in 1×TAE buffer at 100 V for 30 min followed by staining with EB (ethidium bromide, 5 µg/mL). Then, the gel was imaged by UV illumination with 312 nm, and photographed by Gel Imaging system.

Biocompatibility assay.

To assess the cell viability after different dosage administration of CNPs, standard MTT (3-(4,5-dimethylthiazol-2-yl)-2,5-diphenyltetrazolium bromide) assay was performed. Corresponding to transfection experiments, the cell viability assay was performed on four types of cell lines: DC2.4 cell line, RAW264.7 cell line, Hela cell line, HEK-293T cell line. The cells were seeded at the density of 1×10^{4} cells/well in 96-well plates for 24 h. After 24 h, the culture medium was replaced by fresh medium containing CNP particles for 24 h co-incubation. The setting concentrations of CNP were, respectively, 100 μg/mL, 200 μg/mL, 300 μg/mL, 400 μg/mL, 500 μg/mL, and the control group was added into PBS in equal volume with the experimental groups. After 24 h co-incubation, all groups were washed twice with PBS and the medium was replaced with 100 μL fresh medium containing MTT (1 mg/mL) for 4 h incubation at 37 ^{o}C in the incubator. Afterward, the medium was removed gently and 100 μL DMSO was added into each well. After full dissolution, the absorbance of all groups at the wavelength of 490 nm was measured by Biotech SYNERGY H1 microplate reader (Bio Tek, U.S.A.).

Transfection feasibility assay.

mGFP-NP nanocomplexes were prepared with final concentration of 0.5 μM EGFP mRNA and 20 μg/μL DNA-NP-20 at 4 ^{o}C. The nanocomplexes were then heated to 37 ^{o}C to deswell for further use. From the experiment of RNase H-responsive release of mRNA in DNA-NPs, 20 U/mL RNase H could achieve complete release of mRNA from DNA-NPs. 4.4 μL nanocomplexes solution, 0.6 μL RNase H reaction buffer (10×), 0.6 μL RNase H (200 U/mL), and 0.4 μL RNase-free water were gently mixed to make up the reaction volume to 6 μL. The RNase H-cleavage-reaction system was incubated at 37 ^{o}C for 30 min. After centrifugation and purification using EasyPure RNA Purification Kit (TransGen Biotech, Beijing), the purified product containing shortened-polyA mRNA was collected. Transfectional activity of shortened-polyA mRNA was evaluated using DC2.4 cells. DC2.4 cells, seeded at the density of 2×10^5 cells/well in 6-well plates, were cultured in RPMI 1640 medium containing 10% FBS and 1% penicillin/streptomycin. After 24 h of incubation, the medium was removed and the well was washed with PBS when the cell confluence reached 80%. The purified product containing shortened-polyA mRNA was complexed with LipofectamineTM 3000 Transfection Reagent (lipo3000) (Thermo Fisher Scientific). The fresh medium containing lipo3000/mRNA complexes was added into DC2.4 cells for 24 h transfection. The transfected cells were imaged by Biotech Ti-E inverted fluorescence microscope (Nikon, Japan).

HACC modification on the surface of mGFP-NP.

To provide stronger protection against RNases and proper zeta potential for better cellular uptake, quaternary chitosan-2-Hydroxypropyltrimethyl ammonium chloride chitosan (HACC) was applied to modify the surface of mGFP-NPs. mGFP-NPs were prepared at a dose of 1.1 μM EGFP mRNA and 400 μg/mL DNA-NPs as described above. To optimize the cationic HACC density on the particle surface, three different concentrations of HACC were added to the mGFP-NP solution in equal volume: 0.5 mg/mL, 1 mg/mL, 2 mg/mL with mass ratios (mGFP-NP:HACC) of 34, 17, 8.5, respectively. After the complexation process, mGFP-CNP at final DNA-NP concentration of 200 μg/mL in RNase-free water was subjected to DLS measurement using Zetasizer Nano ZS90 (Malvern, U.K.) equipped with 90° optics and a He-Ne Laser (4.0 mW, 633 nm). Three measurements were conferred for each sample, and the average hydrodynamic diameters and zeta potentials were presented.

In vitro transfection of four types of cell lines.

Transfection experiments were conducted on HEK-293T cell line, Hela cell line, RAW264.7 cell line, DC2.4 cell line. HEK-293T cells, Hela cells and RAW264.7 cells were incubated in Dulbecco's modified Eagle's medium (DMEM), immortalized DC2.4 cells were incubated in RPMI 1640 medium. Different cell lines were seeded at a density of 2×10^5 cells/well in confocal dishes and incubated for 24 h at 37 ^{o}C. Afterward, the medium was replaced by fresh serum-free culture medium with the addition of mGFP-CNP. The dosage of EGFP mRNA was 4 μg per well. After 4 h incubation, FBS was added to the cells at a final concentration of 10% and the cells were under another incubation for 20 h. The cells were washed with PBS and were fixed with 4% formaldehyde, the nuclei were stained with 1 μg/mL 4',6-diamidino-2-phenylindole (DAPI). Subsequently, the EGFP expression was observed by confocal microscopy (LSM800 with Airycan, Zeiss, Germany). To study the intracellular presence status of mRNA molecules after 24 h transfection, the Cy5-mGFP-CNPs were introduced into DC2.4 cells following the same transfection procedure as described above and the cells were imaged with CLSM.

Evaluation of cellular uptake.

Immortalized DC2.4 cells were applied to study the cellular internalization of mGFP-CNP. For visualizing the cellular uptake, Cy5-labeled mRNA was prepared using Label IT nucleic acid labeling kit (Mirus, USA) following the manufacturer's protocol and the capturing sequence TAMRA-PolyT was purchased from Sangon Biotech Co., Ltd. (Shanghai). DC2.4 cells were seeded in confocal dishes at a density of 2×10^5 cells/well and incubated for 24 h prior to experiments. They were then treated with mGFP-TAMRA-CNP at a dose of 4 μg mRNA/well over different time periods: 30 min, 1 h, 2 h, 4 h and visualized by confocal microscopy (LSM800 with Airycan, Zeiss, Germany). The mean fluorescence intensity per cell each group was assessed by image J (n=15 cells). For direct internalization evaluation of mRNA, flow cytometry was performed to determine the percentage of Cy5-positive cells compared to non-transfected cells employing a NovoCyte Advanteon (Agilent Technologies). Briefly, HACC-coated DNA-NPs packaging with 2 μg Cy5-mRNA were diluted in serum-containing RPMI 1640 culture medium and incubated over different time periods (2 h, 4h) with 2×10^5 cells seeded in a 6-well plate 24 h prior to the nanoparticles addition. Cells were then washed twice with 500 μL of fresh PBS, followed by suspension of cell precipitation

with another 500 μL PBS. After filtration using 70 μm membrane filter, the cells were applied to quantify the percentage of Cy5-positive cells analyzed by flow cytometry.

Endosomal escape of mRNA facilitating by CNPs.

The experiment was performed on DC2.4 cells to analyze the ability of CNPs to facilitate the endosomal escape. The cells were plated onto the confocal dishes at a density of 2×10^5 cells/well. 24 hours later, the culture medium was replaced with 1 mL Opti-MEM™ Reduced Serum Medium (Thermo Fisher Scientific) containing Cy5-mGFP-CNPs at a dose of 2 μg mRNA/well. After 1 h incubation at 37 ^{o}C, the medium was discarded and cells were washed twice with PBS, followed by addition of 1 mL fresh RPMI 1640 medium. The cells were incubated for another 2 h, 4 h, 6 h. 1 h before the samples were collected, LysoTracker Green DND-26 (Yeasen Biotechnology, Shanghai) was added into the medium following the manufacturer's instruction to stain the lysosomes with green tag. Nuclei were stained blue with DAPI. Then, the intracellular distribution of Cy5-mGFP-CNPs was visualized by confocal laser scanning microscope. The white arrows pointed the mRNA molecules that successfully escaped from lysosomes after 6 h intracellular trafficking.

Intracellular release of mRNA.

To facilitate the detection, the FAM-labeled PolyT (Sangon Biotech, Shanghai) was applied to synthesize the DNA-NPs and then Cy5-mRNA was packaged to finally obtain the Cy5-mGFP-FAM-NPs. Prior to the experiment, DC2.4 cells were seeded at a density of 2×10^5 cells/well in confocal dishes. After 24 h incubation, the medium was replaced with 1 mL Opti-MEM™ Reduced Serum Medium (Thermo Fisher Scientific) containing Cy5-mGFP-FAM-NPs (2 μg Cy5-mRNA per well). 1 h incubation later, the medium was replaced with fresh RPMI 1640 medium following by 6 h culture. The intracellular release of Cy5-mRNA (red) from FAM-DNA-NPs (green) was recorded with CLSM. The red arrows indicated the mRNA molecules successfully separating from the DNA-NP vehicles.

***In vivo* biodistribution imaging.**

The animal experiments were approved by ethics committee of Tianjin University in compliance with the Animal Management Rules of the Ministry of Health of the People's Republic of China (approval number: TJUE-2020-070). The biodistribution of the Cy5 labeled mRNA-CNP (Cy5-mRNA-CNP) in vivo was explored in BABL/C mice. The

BABL/C mice were treated with intramuscular injection of Cy5-mRNA-CNP. After 24 h intramuscular administration of Cy5-mRNA-CNP, the biodistribution of the Cy5-mRNA-CNP was detected with an in vivo imaging system (IVIS Spectrum).

Statistical analysis.

All data were reported as mean ± standard deviation (s.d.) from at least three independent runs. The Student's t-test was used to assess the two-group differences. In all cases, a p-value < 0.05 was considered to be definition of statistical significance. Analyses were performed using OriginPro2018 and GraghPad Prism8.0.

Table S1. DNA sequences used in this study

Name	Sequences (from 5' to 3')
PolyT	5'Acrydite-TTTTTTTTTTTTTTTTTTTTTTTTTTTTTTTTTTTT
TAMRA-PolyT	5'Acrydite-TTTTTTTTTTTTTTTTTTTTTTTTTTTTTTTTTTTT-3'TAMRA
FAM-PolyT	5'Acrydite-TTTTTTTTTTTTTTTTTTTTTTTTTTTTTTTTTTTT-3'FAM
Cy5-PolyT	5'Acrydite-TTTTTTTTTTTTTTTTTTTTTTTTTTTTTTTTTTTT-3'Cy5

Results

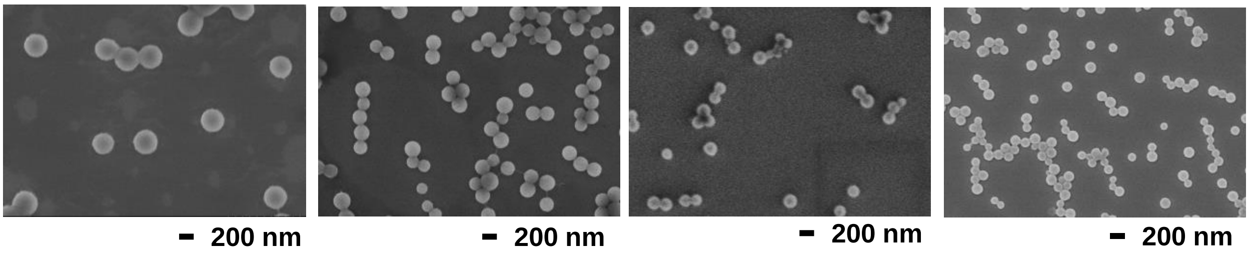

Figure S1. SEM images of DNA-NP-0, DNA-NP-5, DNA-NP-10, and DNA-NP-20.

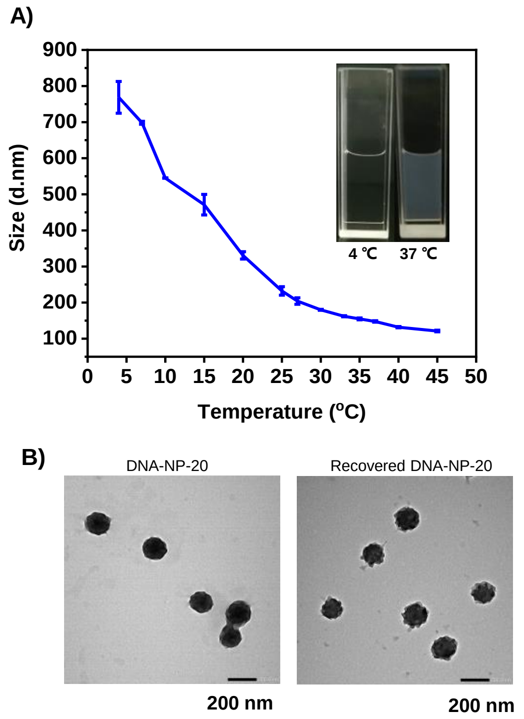

Figure S2. (A) Size of DNA-NP-20 measured by dynamic light scattering (DLS) at different temperatures. Data represented means ± SD, n=3. The inset is the picture of DNA-NP-20 at 4 and 37 °C. (B) The TEM images of DNA-NP-20 and recovered DNA-NP-20.

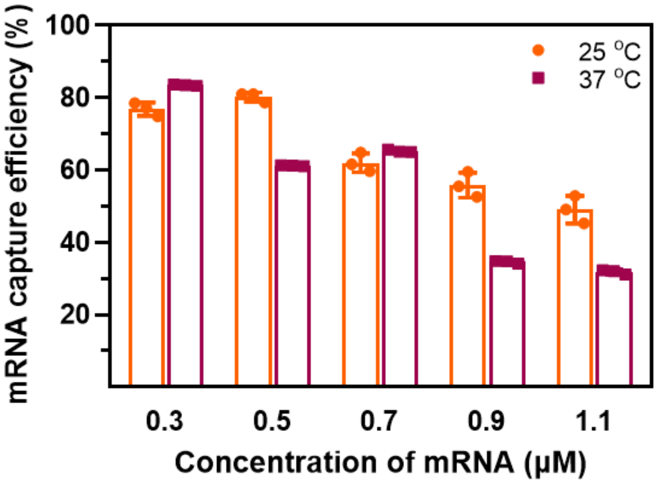

Figure S3. Gray-scale statistics of mRNA capture efficiency according to the gel electrophoresis images of mRNA loading at 25 °C and 37 °C in Figure 2G. Data represented means ± SD, n=3.

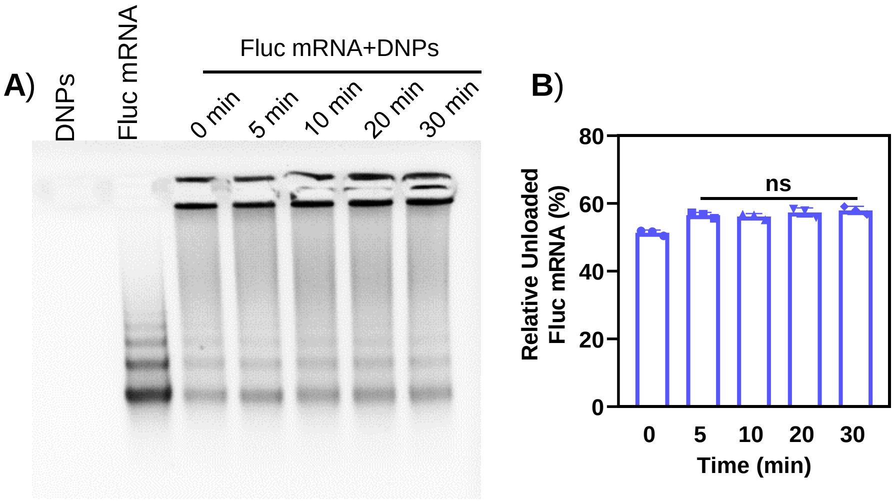

Figure S4. 1% agarose gel electrophoresis image of the loading amounts of Fluc RNA (1929 nt) at different time points.

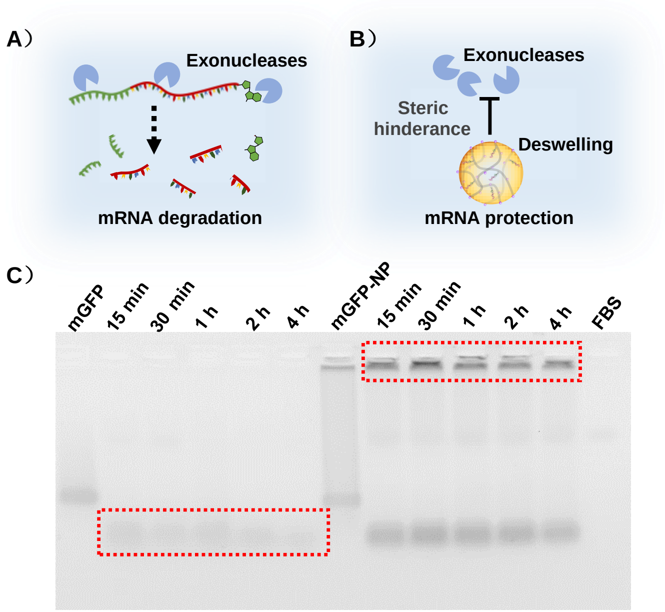

Figure S5. Schematic illustration of mRNA degradation by exonucleases in the physiological environment (A), and DNA-NPs' block effects on RNase attack to mRNA through steric hinderance caused by deswelling (B). 1% agarose gel electrophoresis assay (C) on the protection of DNA-NPs on mRNA in the simulated physiological environment.

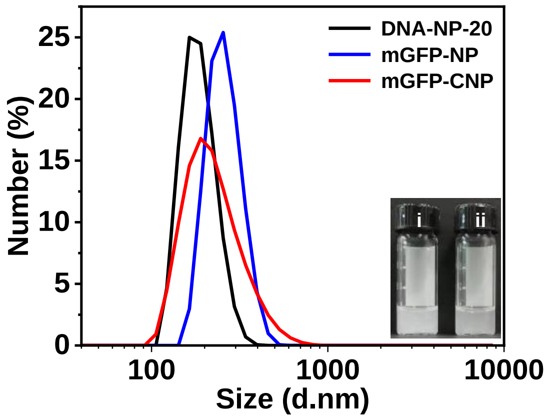

Figure S6. Hydrodynamic size distribution variation of DNA-NP-20, mGFP-NP and mGFP-CNP, the inset is the digital picture of mGFP-NP (i) and mGFP-CNP (ii) at 37 °C.

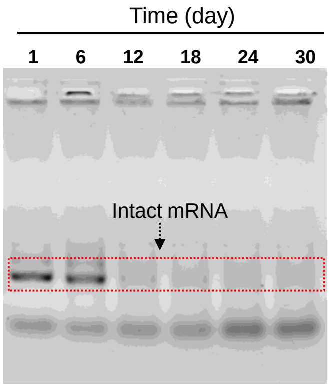

Figure S7. The long-term storage stability of mRNA in mGFP-NP-20 without cryopreservation.

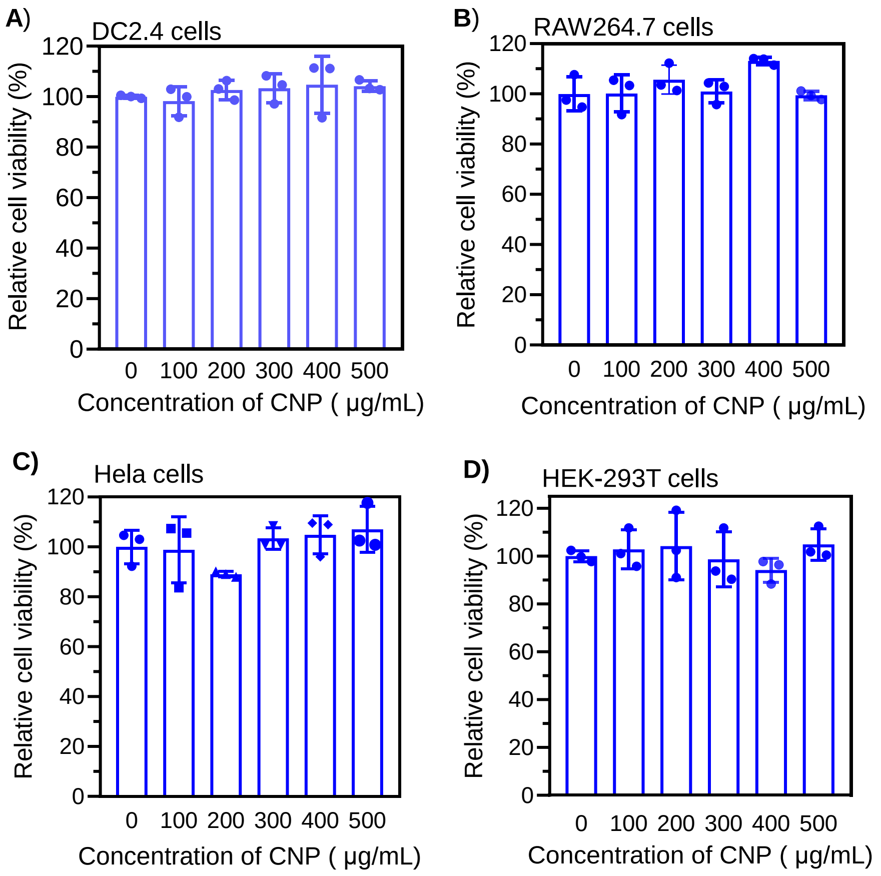

Figure S8. Biocompatibility assay of different concentrations of CNPs *in vitro*: viability of DC2.4 cells (A), RAW264.7 cells (B), Hela cells (C), HEK-293T cells (D) after exposure to different concentrations of CNPs at 37 ^{o}C for 24 h. Data represented means ± SD, n=3.

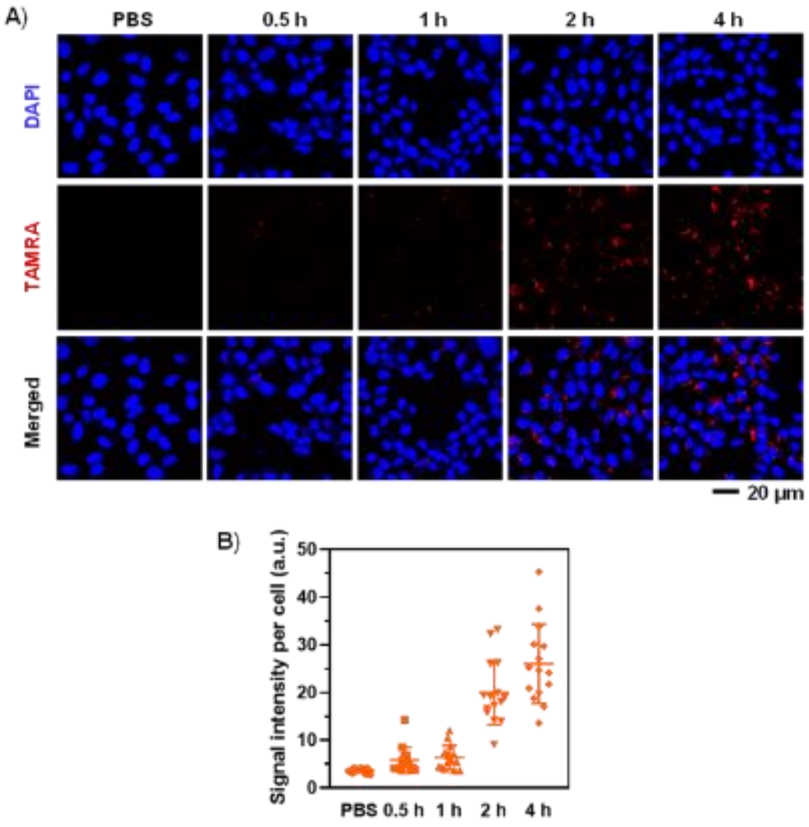

Figure S9. Cellular uptake kinetics of mGFP-TAMRA-CNPs. Confocal microscope images of cellular uptake at different time points (A). Quantitative analysis (B) of fluorescence signal intensity of (A). Error bars represented s.d. (n=15 cells). The experiment was performed on DC2.4 cells.

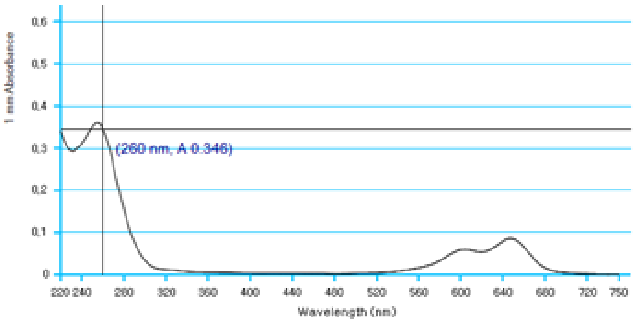

Figure S10. UV-vis absorption spectra of Cy5-mRNA. Verification of mRNA labelling with Cy5 fluorescence tag measured by a nanodrop photometer (Quawell 5000, U.S.A.).

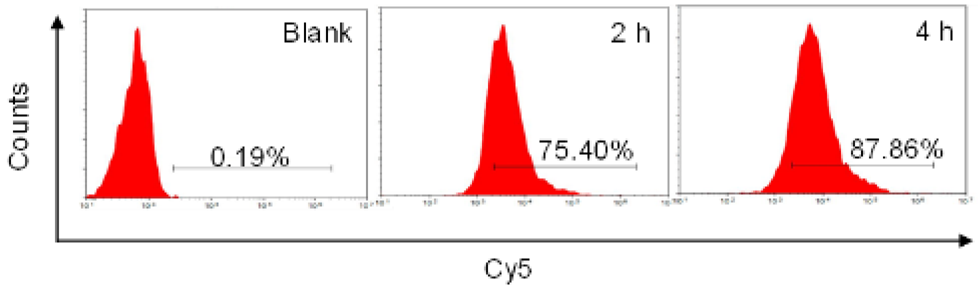

Figure S11. Cellular uptake efficiency at different time points analyzed by flow cytometry. The experiment was performed on DC2.4 cells.

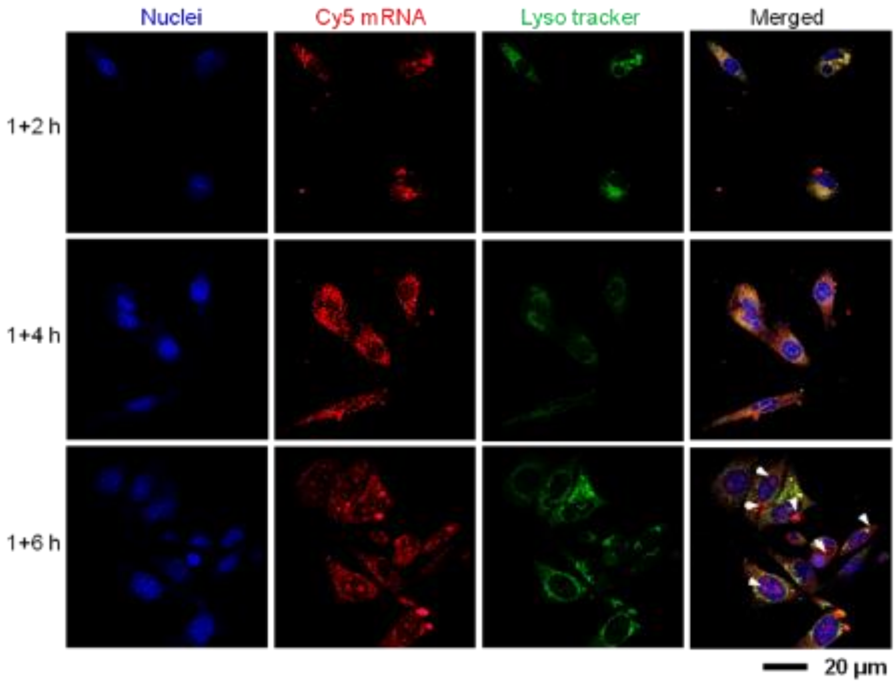

Figure S12. CLSM images of endosomal escape study of Cy5-mGFP-CNPs. The DC2.4 cells were incubated with Opti-MEM medium containing Cy5-mGFP-CNPs at 37 ^{o}C for 1 h, and then the medium was replaced with fresh medium and further incubated for 2 h, 4 h, 6 h, respectively. mRNA molecules were labelled with Cy5 fluorescence tags (red), nuclei were stained with DAPI (blue), lysosomes were tracked with Lysotraker (green).

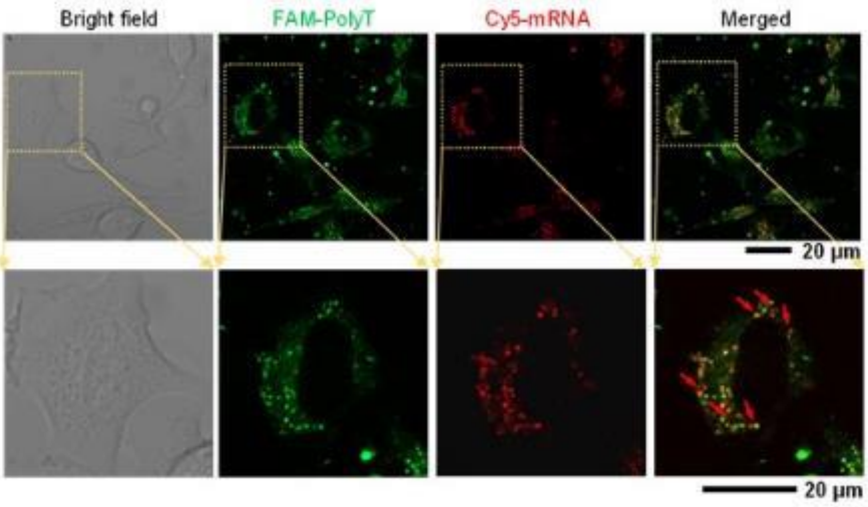

Figure S13. CLSM images of cellular release of Cy5-mRNA from FAM-DNA-NPs. The DC2.4 cells were incubated with Opti-MEM medium containing Cy5-mGFP-FAM-NPs at 37 ^{o}C for 1 h, and then the medium was replaced with fresh medium and further incubated for 6 h. As indicated by the red arrows, there was some obvious separation between the green fluorescence (FAM-Poly-T) and the red fluorescence (Cy5-mRNA), which proved that the mRNAs could not only achieve lysosome escape into the cytoplasm within 6 h, mRNAs also can be released from the capture chain Poly-T in response to intracellular stimulation, allowing the following smooth protein expression.

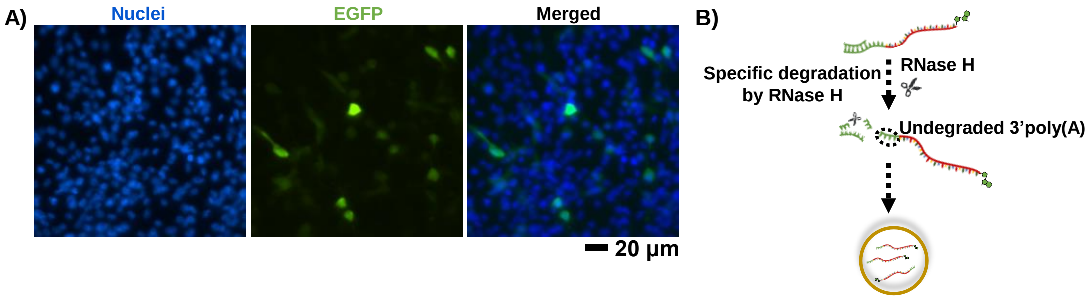

Figure S14. Fluorescence microscope images of transfection of 3' polyA partially degraded EGFP mRNA into DC2.4 cells via lipofectamine3000 reagent (A) and corresponding schematic illustration (B).

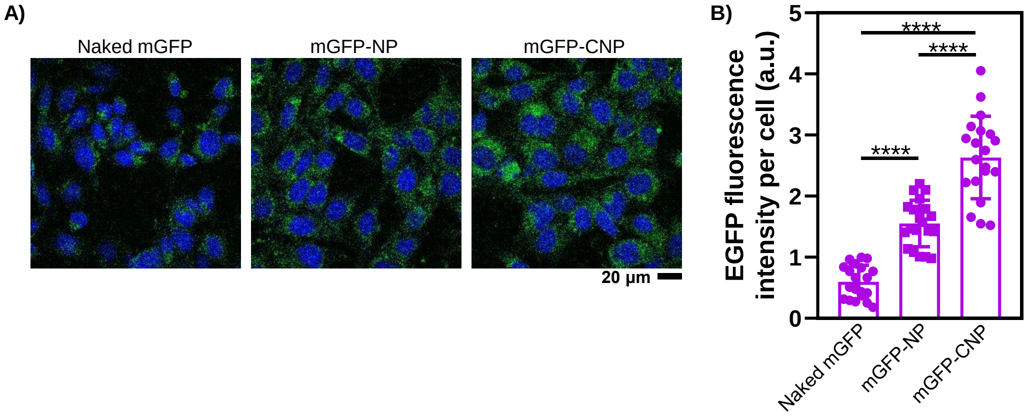

Figure S15. Confocal microscope images (A) of EGFP mRNA transfection into DCs under different formulation treatment: naked mRNA, mGFP-NP, mGFP-CNP. Nuclei were stained blue with DAPI. Quantitative analysis (B) of mean fluorescence intensity of EGFP per cell measured by ImageJ. Error bars represented s.d. (n=20 cells). (****P<0.0001, calculated by unpaired two-tailed t-test). Quantitative statistics of intracellular green fluorescence in the confocal images showed that the transfection level of mGFP-CNP was approximately 1.7 times higher than that of mGFP-NP, and approximately 4.4 times higher than that of naked mGFP. The results demonstrated that the coating of chitosan derivative (HACC) can mediate more protein expression assumably as a result of enhanced cellular uptake.

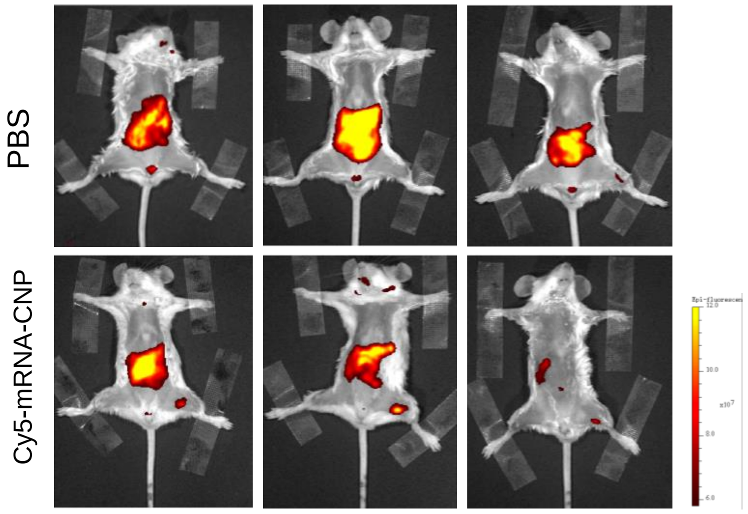

Figure S16. The biodistribution of the Cy5-mRNA-CNP in BABL/C mice after 24 h intramuscular injection.

References

[1] F. Li, W. Yu, J. Zhang, Y. Dong, X. Ding, X. Ruan, Z. Gu, D. Yang, *Nat. Commun.* **2021**, *12*, 1138.
